# Effect of Withania Somnifera on the antioxidant and neurotransmitter status in sleep deprivation induced Wistar rats

**DOI:** 10.6026/97320630016631

**Published:** 2020-08-31

**Authors:** K Suganya, E Kayalvizhi, R Yuvaraj, M Chandrasekar, U Kavitha, K Konakanchi Suresh

**Affiliations:** 1 Meenakshi Medical College Hospital and RI, Physiology, India; 2 Bharath University, India; 3 Sri Devaraj Urs Medical College, Physiology, India

**Keywords:** Sleep Deprivation (SD), Withania Somnifera (WS), Modified Multiple Platform method (MMPM), Oxidative stress parameters, Neurotransmitter

## Abstract

Sleep is normally a period of relaxation and repair, important for the maintenance of physiological homeostasis and psychological balance. "Globally, millions of people experiences sleep deprivation daily". Sleep deprivation (SD) impairs cognitive functions, decreases anti-oxidative defense and induces neuronal changes. Withania somnifera (WS), commonly known as an "Indian Ginseng" has broad therapeutic applications, including anti-inflammatory activities, actions on immune system, circulatory system, central nervous system etc., The study is aimed to assess effect of Withania somnifera on antioxidant status and neurotransmitter level in sleep deprivation induced male Wistar albino rats. The study was done in the Department of Physiology, Meenakshi Medical College and Hospital, Enathur, Kanchipuram. 24 male adult Wistar rats weighing 120-150g were used for the study. They were divided into 4 groups with 6 animals in each group. (Group I - cage control, Group II - large platform control, Group III - sleep deprived group and Group IV – WS treated SD group). Animals were deprived sleep for one week using a modified multiple platform method. Oxidative stress parameters and antioxidant enzymes were measured using spectrophotometry. Neurotransmitters such as dopamine and serotonin concentration in the serum were measured by ELISA method. There was a marked (by one-way ANOVA test) decrease observed in the antioxidants enzymes in the cortex of both large platform control and sleep deprivation induced group. The group treated with W. somnifera root extract significantly reduced the free radical production and lipid peroxidation with simultaneous increase in the level of antioxidant enzymes compared to the untreated group. Also in our study the concentration of dopamine and serotonin was found to be significantly reduced (p < 0.05) in sleep deprived (SD) and large platform control group when compared to cage control group. Whereas the group treated with W. somnifera (400mg/kg b.wt) increased the neurotransmitter levels significantly. Withania somnifera proved to be an effective therapeutic agent by maintaining the antioxidant status and neurotransmitter levels.

## Background

Sleep is normally a period of relaxation and repair, important for the maintenance of homeostasis and psychological balance [1]. It is a physiological phenomenon playing a crucial role in cognition, development of neuronal cells, and maintenance of cardio vascular functions, temperature, water balance and acid base balance of our body. Sleep loss is considered as a health risk that contributes to several disease processes as a result of neurochemical alterations [2]. "Globally, millions of people experiences sleep deprivation daily". Work pattern exposure to artificial light, loud noise, interactive activities including cell phones, television and internet use; all influences normal sleep The population exposed to sleep deprivation is constantly increasing in this modern society. "Work pattern; exposure to artificial light; loud noise; interactive activities, including cell phone, television, and Internet use; all influence normal sleep. The population exposed to sleep deprivation is increasing constantly in modern society".

Sleep deprivation influences the body functions by affecting brain as well as the integumentry systems. People who are deprived sleep experiences behavioral alterations and mood disturbances certainly [3]. The rationale behind this is the imbalance in the secretion of brain neurotransmitters due to interrupted trans- synaptic communication between the amygdala, limbic cortex and pre frontal cortex [4]. Sleep deprivation induces oxidative stress [5] and decreases the anti-oxidative defense mechanism [6]. Oxidative stress develops by free radicals generation and formation of non-radical derivatives of oxygen and nitrogen molecules [7]. These products are generally involved in normal cell regulation and signal transduction and an imbalance between their generation and the antioxidant defense system results in oxidative stress [8].

"Withania somnifera originated from the dry regions of South Asia, such as India, Pakistan, Afghanistan, where it is widely cultivated; and now grows as well in South Africa and other countries [9]. "Withania somnifera originated from the dry regions of South Asia, such as India, Pakistan, Afghanistan, where it is widely cultivated; and now grows as well in South Africa and other countries" [9]. "Withania somnifera originated from the dry regions of South Asia, such as India, Pakistan, Afghanistan, where it is widely cultivated; and now grows as well in South Africa and other countries [9]. Withania somnifera originated from the dry regions of South Asia such as India, Pakistan, Afghanistan where it is widely cultivated; and now grows as well in South Africa and other countries [9]. It is a member of Solonaceae family and known by many names such as "Ashwagandha", "Queen of Ayurvedha", "Indian Ginseng" etc. and has been an important herb in indigenous medicinal system for more than 3000 years [10]. It has broad therapeutic applications, including anti-inflammatory activities, actions on immune system [11], circulatory system [12], anti diabetic [13], on central nervous system [14] etc. These therapeutic applications of W. somnifera are related to the presence of alkaloids and lactones found at different levels of plant parts. The roots and leaves of W. somnifera were reported to have a richer source of withanolides and related compounds [15]. These withanolides have been found cytotoxic to cancer cells and possess immunomodulatory, neuroprotective and antioxidant properties [16,17].

## Materials and Methods:

The study was done in the Department of Physiology, Meenakshi Medical College and Hospital, Enathur, Kanchipuram. Proper Ethical clearance was obtained from the CPCSEA (IAEC No: 007/2017). Twenty-four male 8- week-old Wistar rats weighing 120-150g were used for the study. They were divided into 4 groups with 6 animals in each group. (Group I-cage control, Group II-large platform control, Group III-sleep deprived group and Group IV-WS treated SD group). Animals were deprived sleep for one week using a modified multiple platform method [18]. Oxidative stress parameters and antioxidant enzymes were measured using standard methods21. Neurotransmitters such as dopamine and serotonin concentration in the serum of both control and sleep deprived rats were measured by ELISA kit. Statistical analysis was done by one-way ANOVA and Duncun's multiple range test.

## Sleep Deprivation Technique:

Modified Multiple Platform Method18

The rats were deprived of sleep for one week using modified multiple platform method. The rats were placed on the circular platforms (7cm in diameter), which are fixed inside a water tank filled with water of approximately 1cm. Though each group has 6 rats, around 12 circular platforms were fixed inside the water tank. This allowed the rats to move around freely inside the tank by jumping from one platform to another. When the rat falls asleep it loses muscle tone and falls off from the platform into the water, awaking from sleep, then climbs back up. Thus, sleep deprivation was achieved by depriving the rats of sleep. Food and water were provided ad libitum by placing chow pellets and water bottles on a mesh located at the top of the tank. The water in the tank was changed daily throughout the SD period. After the adaptation period, the rats were placed in the MMPM and subjected to total sleep deprivation for one week. Large Platform Control group rats (Group II) were left free on a large platform (14 cms) in the same environment where sleep deprivation was performed.

## Ethanolic extract of Withania somnifera:

Air dried and powdered root material of W.Somnifera was extracted with 95% ethanol for 12 hours in Soxhlet extractor. The obtained extract was concentrated using rotary vacuum evaporator at 40- 60°C. The concentrated semisolid extract was stored in refrigerator at 2-8°C till further use. The extract was dissolved in DMSO (Dimethyl sulphoxide) and administered orally to animals for 30 days with a dosage of 400 mg/kg bw [19,20].

## Assessment of Serum Neurotransmitters in the control and sleep deprived rats:

Neurotransmitters such as dopamine and serotonin concentration in the serum of control and sleep deprived rats were measured by rat dopamine and serotonin kits obtained from bioassay technologies (Shanghai Korain Biotech Co., Ltd, #1008 Junjiang Inter Bldg, 228 Ningguo Rd, Yangpu Dist, Shanghai, China 200090). Results are expressed as ng/ml.

## Estimation of Lipid peroxidation and reactive oxygen species:

Estimation of Lipid peroxidation, Hydrogen peroxide and Hydroxyl radical generation was done using Spectrophotometry [21].

## Estimation of antioxidant enzymes:

Estimation of superoxide dismutase (SOD), Assay of Catalase (CAT), Assay of Glutathione peroxidase (GPX), Assay of Glutathione Reductase (GR), Glutathione-S-Transferase (GST) and Assay of Reduced Glutathione (GSH) were measured using standard methods [21] by Spectrophotometry.

## Statistical analysis:

The data obtained were subjected to statistical analysis using one-way analysis of variance (ANOVA) and Duncan's multiple range test to assess the significance of individual variations between the control and treatment groups using a computer based software (Graph Pad Prism version 5). In Duncan's test, the significance was considered at the level of p < 0.05.

## Results

**Figure 1:**
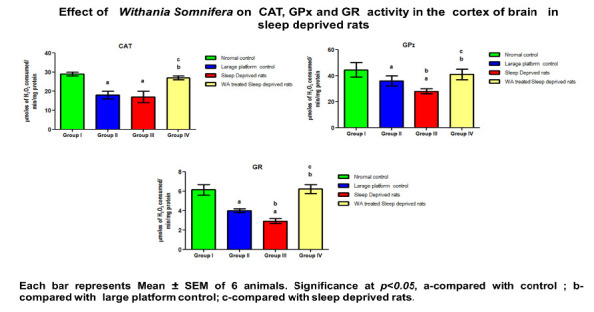
Effect of Withania Somnifera on CAT, GPx and GR activity in the cortex of brain in sleep deprived rats

**Figure 2:**
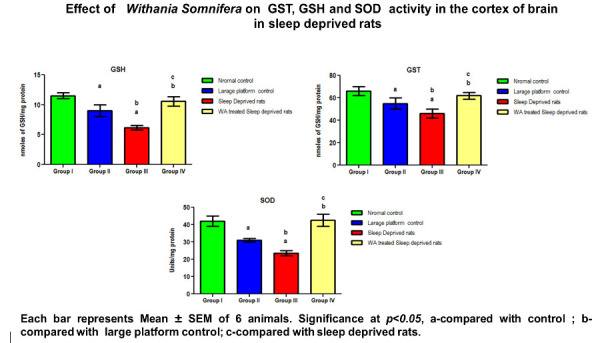
Effect of Withania Somnifera on GST, GSH and SOD activity in the cortex of brain in sleep deprived rats

**Figure 3:**
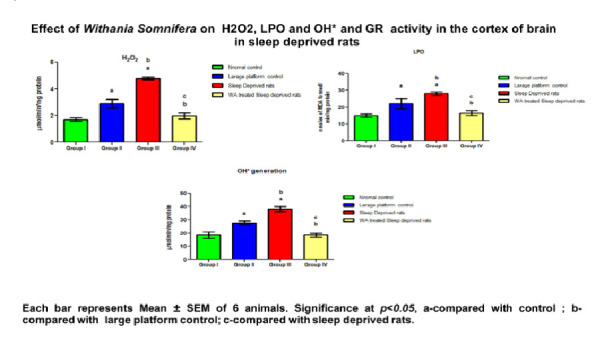
Effect of Withania Somnifera on H2O2, LPO and OH+ activity in the cortex of brain in sleep deprived rats

**Figure 4:**
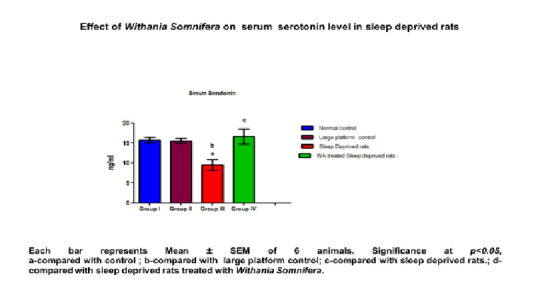
Effect of Withania Somnifera on serum serotonin level in sleep deprived rats

**Figure 5:**
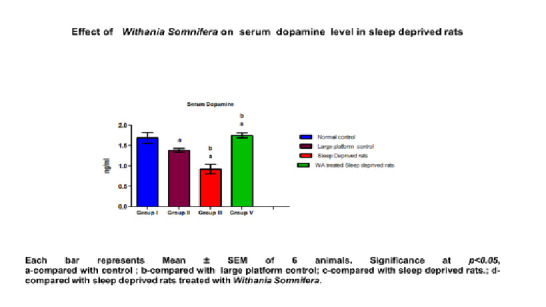
Effect of Withania Somnifera on serum dopamine level in sleep deprived rats

There was a marked decrease observed in antioxidants enzymes (SOD, CAT, GPx, GST, GR and GSH) in the cortex of large platform control and sleep deprivation induced rats. W.somnifera treatment to SD rats, significantly increased the level of antioxidant enzymes compared to the untreated group (Table 1; Figure 1 and Figure 2). Free radicals and lipid peroxidation estimated in the cortex was found to be significantly (p<0.05) elevated in large platform control group and sleep deprived rats when compared with cage control rats. Treatment with W. somnifera root extract significantly (p < 0.05) decreased the free radical production and lipid peroxidation (Table 2; Figure 3). In the sleep deprived (SD) and large platform control rats, the concentration of dopamine and serotonin were found to be significantly-reduced (p < 0.05) when compared to control rats. Pretreatment with W.somnifera (400mg/kg b.wt) showed increased concentration of neurotransmitters significantly (Table 3; Figure 4 and Figure 5).

## Discussion

Sleep occupies approximately one-third of a person's lifetime. Modern society increases variety of complex activities tending to push sleep into background. This result in impaired concentration, altered behavior, reduces the quality of life and failure to complete routine activities. The findings of our study reveal an elevated free radical production and lipid peroxidation levels in SD rats owing to excess nitric oxide (NO) production [26]. Along with these decreased levels of enzymatic and non-enzymatic antioxidants in SD animals signifies its excess utilization by the cells. The above-mentioned changes results in oxidative burden leading to pathogenesis of several disease states in SD. Since brain tissues consist of a high content of polyunsaturated fatty acids and one of the important consequences of oxidative stress is peroxidation of membrane lipids, this reaction produces marked damage to the structure and function of the brain tissues [27]. Significant elevation in antioxidant levels with concomitant decrease in LPO and oxidative stress radicals in our study might be due to the active principles of W. Somnifera, sitoindosides VII-X and withaferin A (glycowithanolides) [28] which has a protective role against the oxidative stress induced neuronal damage in sleep deprivation.

The levels of neurotransmitters (Dopamine and Serotonin) were significantly decreased in large platform control and sleep deprived rats when compared with cage control rats. The elevated pro-inflammatory cytokines in SD rats [22] play a key role by affecting the metabolism of monoamine neurotransmitters (dopamine, serotonin, and nor-epinephrine) and decreasing its availability, synthesis and increasing the uptake. These changes have a potential effect on inflammation, which develops chronic stress leading to behavioral changes22. In addition, the reduction in the 5-HT levels might also be due to the degeneration of dorsal raphe nucleus, which secretes serotonin. The 5-HT neurons are vulnerable to cell death as they are stress sensitive and contain glucocorticoids and NMDA receptors, which are activated by stress, induced corticosterone activity leading to apoptosis [23].

Similarly, the decline in the dopamine levels in SD rats might be due to Oxidative stress, mitochondrial dysfunction and deregulated intracellular calcium levels with alpha-synuclein aggregation causing degeneration of dopamine secreting cells [24]. Hence it is clear that sleep has a strong influence on DA pathway and the involvement of dopaminergic system on cognitive functions. In our study, the group treated with Withania Somnifera significantly increased the neurotransmitter levels. The bioactive chemical principles, glycol-withanolides may exert a role by preventing the degeneration of neurons involved in the secretion of neurotransmitters [25].

## Conclusion

In our present study, sleep deprivation altered brain antioxidants as well as neurotransmitters in male Wistar rats and hence proved to be potent stressor. Withania somnifera, a popular herb exhibited neuro-protective role through normalization of neurotransmitters and restoration of antioxidants in the cortex of sleep deprivation-induced Wistar rats. Hence, W.somnifera root extract can be used as one of the potential therapeutic drug for the treatment of sleep deprivation-induced neuronal diseases.

## Declaration on Publication Ethics:

The authors state that they adhere with COPE guidelines on publishing ethics as described elsewhere at https://publicationethics.org/. The authors also undertake that they are not associated with any other third party (governmental or non-governmental agencies) linking with any form of unethical issues connecting to this publication. The authors also declare that they are not withholding any information that is misleading to the publisher in regard to this article.

The authors are responsible for the content of this article. The Editorial and the publisher has taken reasonable steps to check the content of the article with reference to publishing ethics with adequate peer reviews deposited at PUBLONS.

## Figures and Tables

**Table 1:** Effect of W.somnifera root extract on antioxidant enzyme activity in the cortex of control and sleep deprived rats

Parameters	Normal control	Large platform control	Sleep Deprived rats	W. Somnifera treated Sleep deprived rats	P Value
CAT	29±1	18±2 a	17 ±1a	27 ± 1a,b,c	<0.0248
GPx	44.5 ±5.5	36 ±4.a	26± 2 a,b	41 ±4 a,b,c	<0.0519
GR	6.15± 0.55	4 ±0.2 a	1.23± 0.26 a,b	6.20 ±0.6 a,b,c	<0.0096
GSH	11.50 ±0.50	9 ±0.80	6.4 ±0.36 a,b	10.5 ± 0.07 a,b,c	<0.0212
GST	66 ±4	55 ±5	46 ±4a	62 ± 3 a,b,c	<0.0456
SOD	42±3	31 ±1	23 ±1a,b	42± 3.5 a,b,c	<0.0142
P value <0.05 statistically significant.

**Table 2:** Effect of W.somnifera root extract on hydrogen peroxide, hydroxyl radical and lipid peroxidation in the cortex of control and sleep deprived rats.

Parameters	Normal control	Large platform control	Sleep Deprived rats	W.somnifera treated Sleep deprived rats	P Value
H2O2	1.69±0.13	2.89 ±0.3a	4.78 ±0.11a,b	1.97 ±0.22a,b,c	<0.0017
LPO	15± 1	22 ±2a	28±1 a,b	16 ±1 a,b,c	<0.0228
OH	18±2.5	27 ±1.5 a	38±2 a,b	18 ±1.5 a,b,c	<0.0054
P value <0.05 statistically significant.

**Table 3:** Effect of W.somnifera root extract on neurotransmitters in the serum of sleep deprivation-induced male rats.

Parameters	Normal control	Large platform control	Sleep Deprived rats	W.Somnifera treated Sleep deprived rats	P value
Dopamine (ng/ml)	1.68±0.13	1.38 ±0.044	0.923 ±0.078a,b	1.77 ± 0.064a,b,c	< 0.0241
Serotonin (ng/ml)	15.69 ±0.690	15.54 ±0.57	9.496± 0.620 a,b	16.59 ±1.07 a,b,c	<0.0570
P value <0.05 is statistically significant.
